# Evaluation of fatigue progression during overhead tasks and the effects of exoskeleton assistance

**DOI:** 10.1017/wtc.2025.10008

**Published:** 2025-06-11

**Authors:** Seemab Zakir, Lorenzo Grazi, Francesco Giovacchini, Nicola Vitiello, Emilio Trigili, Simona Crea

**Affiliations:** 1 The BioRobotics Institute, Scuola Superiore Sant’Anna, Pisa, Italy; 2 Department of Excellence in Robotics & AI, Scuola Superiore Sant’Anna, Pisa, Italy; 3 IUVO S.r.l., Pontedera, Italy

**Keywords:** exoskeletons, biomechanics, human-robot interaction

## Abstract

Upper-limb occupational exoskeletons reduce injuries during overhead work. Previous studies focused on muscle activation with and without exoskeletons, but their impact on shoulder fatigue remains unclear. Additionally, no studies have explored how exoskeleton support levels affect fatigue. This study investigates the effects of assistive profiles on muscular and cardiovascular fatigue. Electromyographic (EMG) and electrocardiographic signals were collected to compute EMG median frequency (MDF), heart rate (HR), and heart rate variability (HRV). Fatigue was assessed using three MDF and HR metrics: relative change ( 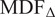 , 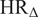 ), slope ( 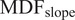 , 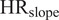 ), and intercept ( 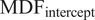 , 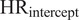 ) of the linear regression. Results showed 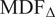 decreased 64% (*p* = 0.0020) with higher assistance compared to no exoskeleton; 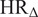 decreased 40% (*p* < 0.0273) with lower assistance, 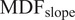 decreased up to 67% (*p* = 0.0039) and 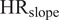 by 43% (*p* < 0.0098) with higher and medium assistance. HRV metrics included root mean square of successive differences (RMSSD) and low-frequency to high-frequency power ratio (LF/HF). RMSSD indicated parasympathetic dominance, while rising LF/HF ratio suggested physiological strain. Findings support occupational exoskeletons as ergonomic tools for reducing fatigue.

## 1. Introduction

Work-related musculoskeletal disorders (WMSDs) are the main cause of occupational illness in Europe, affecting around 30–56% of the working population, with shoulder injuries being particularly prevalent among industrial workers (De Kok et al., 2019). The economic implications of these disorders are profound, accounting for approximately 2.5% of the EU’s gross domestic product due to productivity losses. These losses cause significant costs for employers, including direct compensation and indirect costs like lost wages, production disruptions, recruitment expenses, and healthcare for affected workers (Bevan, 2015). Working in these awkward postures imposes complex and concurrent stresses on the upper extremities, leading to increased muscular contraction, fatigue, and soreness, due to repetitive or prolonged muscle contractions without adequate rest (Parent-Thirion et al., 2017; Umer et al., 2020). Indeed, working overhead has been linked to a multitude of negative physiological and biomechanical consequences, with increased intramuscular pressure, impaired circulation, increased muscle activity, and fatigue development (Dickerson et al., 2011). Concurrently, these working activities can also overload the cardiovascular system, causing a general feeling of tiredness due to an increase of overall physical fatigue (Feng et al., 2023).

In this context, occupational exoskeletons (OEs) are being explored as a mean to alleviate the physical demands on workers and reduce the risk of developing WMSDs (De Looze et al., 2016). OEs are personal assistive devices designed to reduce the physical load on workers performing demanding activities by working in synergy with their users (Monica et al., 2020). OEs are designed to support specific body parts, such as the upper limbs, or improve specific human capabilities by reducing the effort required to perform a work task. OEs for the upper extremities typically target the shoulder joint, providing anti-gravitational support to assist the upper limbs and reduce the biomechanical load on the shoulder joint. According to their actuation principle, these devices can be categorized as passive, active, or semi-active devices, with most commercially available models being passive (Theurel and Desbrosses, 2019; Crea et al., 2021). Active systems use actuators to supply energy, while passive systems store and release the mechanical energy through elastic components; instead, semi-active devices are a tradeoff between active and passive systems. They employ low-power actuators to modulate the behavior of spring-based mechanisms typical of passive devices, offering significant potential by ensuring a certain degree of adaptiveness (Grazi et al., 2020).

Several studies have shown the effectiveness of shoulder OEs in reducing physical strain, in highly controlled laboratory settings, while testing OEs in real-world scenarios (Crea et al., 2021). Typically, these studies analyzed muscle activation in in-lab, simulated, and real industrial settings, where participants perform the same activity with and without the exoskeleton (De Looze et al., 2016; Huysamen et al., 2018; Moyon et al., 2018; Van Engelhoven et al., 2019; Maurice et al., 2020; Pacifico et al., 2020, 2022; Grazi et al., 2024; Ramella et al., 2024). Nevertheless, only a few studies have focused on assessing physical fatigue in conjunction with shoulder exoskeleton use (De Bock et al., 2022; Van Der Have et al., 2022), while no studies have investigated how different support levels provided by an OE influence physical fatigue.

Physical fatigue is typically described as a reduction in the capacity to perform physical work as a function of preceding physical effort (Gawron et al., 2001). It can be related to the activity being performed and caused by, for example, the amount and type of physical load (static or repetitive) and performing work in awkward postures (Bangaru et al., 2022). On the one hand, physical fatigue can be localized to the muscles and defined as the decrease in the maximal force or power production in the muscle in response to its contractile activity (Gandevia, 2001). Muscle fatigue is a commonly experienced phenomenon that limits the capacity to perform physical activities, especially during strenuous or prolonged tasks (Wan et al., 2017). While reducing the muscle’s physical capacity, it can also increase the likelihood of the insurgence of musculoskeletal pain and injury (Larsson et al., 2007; Antwi-Afari et al., 2017). On the other hand, prolonged physical exertions and execution of demanding physical activities can increase the cardiovascular load, thus indicating physical fatigue at the body level (Chen et al., 2023).

Muscle fatigue is typically studied by means of surface electromyography (EMG) measurements, since biochemical and physiological changes in muscles during fatiguing contractions are also reflected in the properties of myoelectric signals recorded on the skin’s surface above the muscles (De Luca, 1984). In the last decades, several approaches have been proposed to measure muscle fatigue through EMG signals, by means of time, frequency, and combined time-frequency methods (Cifrek et al., 2009). Time-domain methods typically involve estimating the amplitude of EMG signals, such as the mean absolute value or root mean square. However, some studies have suggested that EMG amplitude may not reliably predict the level of muscle activation and muscle force when fatigue is present (Dideriksen et al., 2010); thus, these metrics are rarely used on their own as indicators of muscle fatigue. Instead, they are often combined with frequency-domain indicators, such as spectral analysis (Cifrek et al., 2009). These methods rely on the fact that the primary change in EMG signals during sustained contractions is a shift in the signal power spectrum toward lower frequencies; this shift can be quantified using various methods of frequency analyses, such as Fourier-based and parametric-based spectral estimators (Cifrek et al., 2009). Time-frequency methods, such as using short-time Fourier transform and spectrogram, allow to quantify the variations of the EMG signal power spectrum as a function of time. In this respect, Cifrek and colleagues developed a method for analyzing the changes in the median frequency (MDF) of the EMG power spectrum, where the decrease over time of the MDF, calculated from the spectrogram, due to reduced conduction velocity of muscle fibers, was used as an indicator of muscle fatigue (Cifrek et al., 2000).

Cardiovascular fatigue results from the sustained physical workload due to a physical activity and is typically measured through electrocardiography (ECG) (Chen et al., 2023). From a physiological standpoint, oxygen transport during acute fatigue is affected by the autonomic nervous system response; this may be due to the fact that reduced cardiac output, combined with an elevated heart rate (HR), leads to decreased heart rate variability (HRV), which reflects fluctuations in the intervals between consecutive heartbeats (R-R intervals): high HRV following physical exercise indicates a well-balanced autonomic system with greater parasympathetic activity, whereas low HRV may point to sympathetic dominance or stress (Vasquez-Bonilla et al., 2024). HR signals can therefore serve as an indirect measure of worker’s physical effort during tasks with some studies, indicating a progressive increase in HR during high workloads until fatigue occurs (Fisher and Secher, 2019). Additionally, a direct relationship between physical fatigue and HR metrics has been observed (Tran et al., 2009). It has been shown that monitoring HR signals can be an effective tool for assessing the physiological strain of workers (Gatti et al., 2014). The study from (O’Neill & Panuwatwanich, 2013) highlighted the financial losses due to fatigue, amounting to $50,000 annually for a 10-member concrete crew. This underscores the importance of HR monitoring in boosting productivity by identifying when workers are approaching fatigue, allowing for timely interventions (such as rest periods or task modifications).

This study aims to investigate the effect of different levels of anti-gravitational support provided by a semi-active upper-limb exoskeleton on physical fatigue during an overhead screwing task. We hypothesized that, in overhead quasi-static tasks, increases in exoskeleton’s assistance, namely, increases of anti-gravitational support at the shoulder joint, could delay the onset of the physical fatigue both at muscular and cardiovascular level, thus enhancing worker’s endurance with potential impact in reducing the insurgence of work-related MSDs.

## 2. Materials and methods

This study leverages an existing dataset originally collected to validate the effectiveness of a semi-active upper-limb occupational exoskeleton (H-PULSE) during a simulated overhead screwing/unscrewing task (Grazi et al., 2020). In this section, after a recap of the exoskeleton mechatronics and the experimental setup, reported with more details in Grazi et al., 2020), we report the methodology to compute the fatigue-related metrics in all experimental conditions.

### 2.1. Exoskeleton

**Figure 1.**
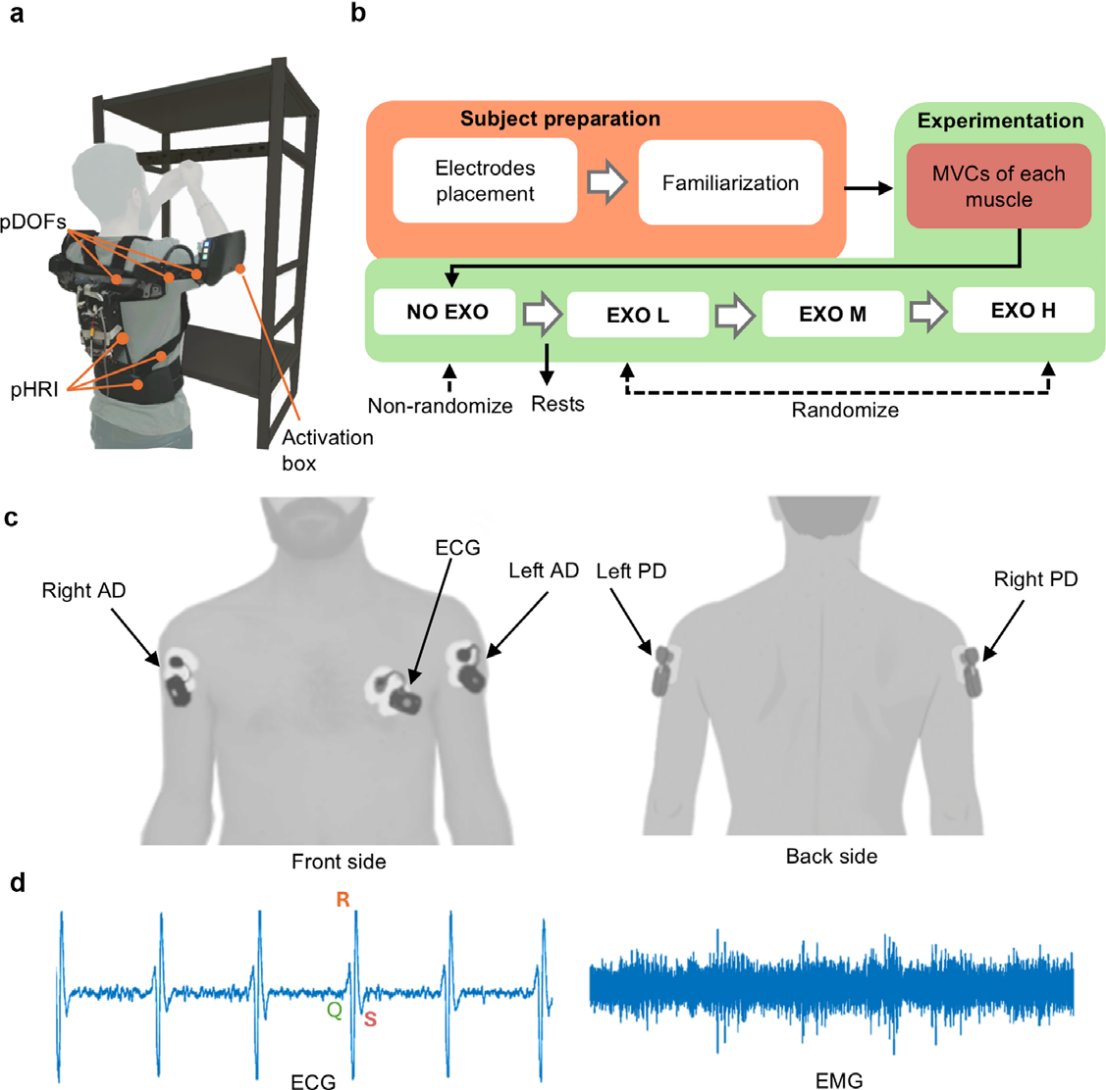
Experimental setup and protocol. (a) The participant wearing the exoskeleton during the experiment. (b) Overview of experimental protocol. (c) Representation of sensor placement. (d) Raw physiological signals.

The H-PULSE exoskeleton integrates a physical Human-Robot Interface (pHRI) in the form of a wearable vest, a passive degrees of freedom chain (pDOFs) that supports shoulder flexion/extension movements, and two actuation boxes (one per arm) that generate the assistive support, through a spring-based mechanism (Figure 1a). The assistive torque depends on the shoulder flexion/extensions (sFE) angle, following an angle-torque relationship like the effect of gravity on a human arm. Specifically, the mechanism is designed to produce maximum torque when the arm is at approximately 90 degrees and zero torque when the arm is parallel to the body. In the latter position, the output torque is zero due to the absence of a lever arm. Within each actuation box, the spring-based mechanism can also be automatically pretensioned using a low-power servomotor coupled with a spindle drive. By changing the pre-tension of the spring mechanism, the peak torque can be varied from approximately 4.5–6 N·m. The spring pre-tension is pre-configured at three discrete levels: low, medium, and high assistance for easy adjustment. Additionally, more precise tuning options are available.

### 2.2. Experimental setup and protocol

Ten right-handed male participants (age: 28.5 ± 2.5 years) took part in the study carried out at The BioRobotics Institute of Scuola Superiore Sant’Anna (Pontedera, Pisa, Italy), following ethics approval by the local Institutional Review Board (approval n. 2/2019). The experimental procedures were conducted in accordance with the principles stated in the Declaration of Helsinki. The participants signed the written informed consent to participate in the study.

The anthropometric parameters of the study population are shown in Table 1; the gravitational torque, 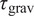 (due to the human arm’s weight), that acts on the human glenohumeral joint is estimated based on the subject’s weight and height, according to equation (1):
(1)

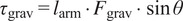

**Table 1.** Anthropometric characteristics of participants

Subject (#)	Height (cm)	Weight (kg)	BMI (kg/m^2^)	Arm length (cm)	Arm weight (kg)	Gravitational force (N)	Gravitational torque (N.m)
1	171	64	21.9	56.9	3.20	31.36	9.46
2	178	62	19.6	59.3	3.10	30.38	9.54
3	178	82	25.9	59.3	4.10	40.18	12.62
4	170	68	23.5	56.6	3.40	33.32	10.00
5	184	78	23.04	61.3	3.90	38.22	12.41
6	165	76	27.9	54.9	3.80	37.24	10.84
7	172	70	23.7	57.3	3.50	34.30	10.41
8	175	75	24.5	58.3	3.75	36.75	11.35
9	173	57	19.04	57.6	2.85	27.93	8.53
10	165	70	25.7	54.9	3.50	34.30	9.99
Mean ±SD	173.1±5.9	70.2±7.8	23.5±2.8	57.6±1.98	3.51±0.4	34.40±3.8	10.51±1.3

*Note*: Average values are reported as mean ± standard deviation.

where 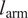 represents the position of the human arm’s center of mass, estimated based on the subject’s height. 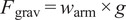 is the gravity force of the human arm, 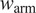 is the arm mass estimated as a percentage of the subject’s mass, and *g* is the gravitational acceleration. Additionally, *θ* denotes the shoulder flexion angle, where 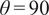 deg represents the condition of maximal gravitational torque (Grazi et al., 2020).

To simulate typical overhead activities that closely approximated industrial workflows, the overhead screwing task was selected as a representative scenario (Schmalz et al., 2019). The experimental task began with a 2-min rest phase, during which participants stood still with their arms parallel to their bodies. Following this, they proceeded to the task phase, where they screwed and unscrewed a screw inserted into a metal support fixed to a shelf. This was performed continuously for 6 min, without any rest breaks or transitions, with both arms raised overhead and shoulders and elbows flexed at approximately 90 degrees. Participants used an Allen key to complete the task at a self-selected, steady pace of screw turn. The experiment included four conditions: one without the exoskeleton (NO EXO condition), representing 0% anti-gravitational support, and three with the exoskeleton, each providing various levels of assistance (EXO L, EXO M, EXO H). To ensure participants were in the same physical state before starting each condition, a 10-min rest break between conditions was provided, which was deemed sufficient to minimize fatigue effects. The assistive conditions (EXO L, EXO M, EXO H) corresponded to different percentages of anti-gravitational support. In the EXO L condition, the exoskeleton provided around 46% (42–47%) of anti-gravitational support to the left side and 47% (42–50%) to the right side; in the EXO M condition, the left side received around 51% (47–53%) of anti-gravitational support and the right side around 53% (42–55%); and in the EXO H condition, the exoskeleton provided around 55% (51–61%) of anti-gravitational support to the left side and 57% (47–60%) to the right side, respectively.

Figure 1 shows the experimental setup, protocol, and sensor placement. Figure 1b represents a participant performing the experiment while wearing the exoskeleton. Figure 1b details the experimental protocol, starting with the preparation phase, which includes placing sensors on the target muscles and familiarizing the participant with the procedure. This is followed by the experimentation phase, where maximum voluntary contractions (MVC) are recorded for each targeted muscle, and the participant performs the experimental tasks. Electromyography (EMG) and electrocardiography (ECG) signals were recorded to measure participants’ muscular and cardiovascular fatigue, respectively. These signals were acquired at 1 kHz using the BTS FREEEMG 1000 (BTS Bioengineering, Milan, Italy). Myoelectric signals from the Anterior Deltoid (AD) and Posterior Deltoid (PD) of both shoulders were collected through pre-gelled bipolar Ag/AgCl surface electrodes (Pirrone & Co., Milan, Italy), placed on the participants’ skin (Figure 1c) following the SENIAM guidelines (Hermens et al., 2000).

To record the ECG signal, an additional sensor was placed over the left Pectoralis Major (PM) muscle (Figure 1c). During the task, the signal-to-noise ratio was deemed sufficient to clearly detect the QRS complex and accurately calculate the HR signal. An example of ECG signal is shown in Figure 1d.

### 2.3. Data analysis

**Figure 2.**
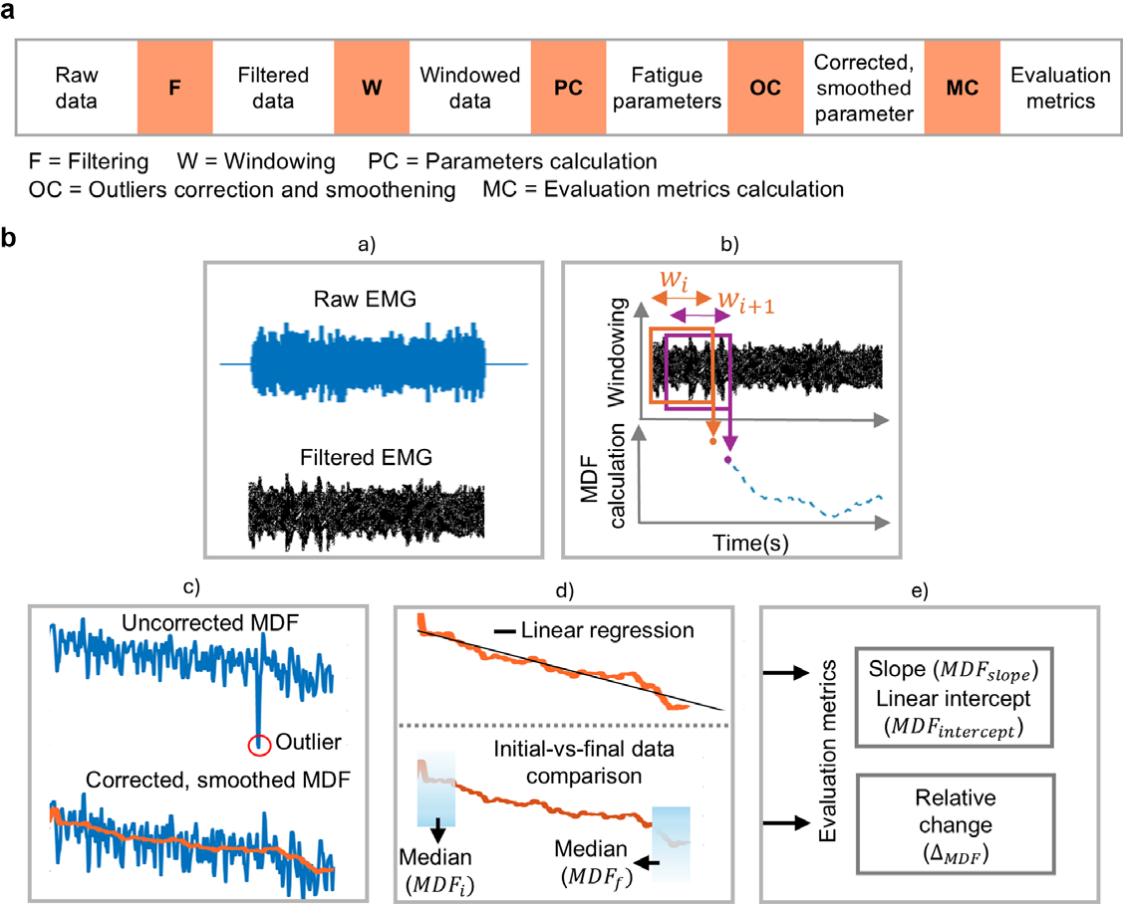
Schematic representation of data processing. (a) Generalized flowchart for processing physiological signals, applicable to both electromyographic (EMG) and electrocardiographic (ECG) data. (b) A graphical representation showing the signal processing steps, with EMG data used as an example: (a) Filtering the EMG signal to remove noise. (b) Dividing the filtered data into windows and calculating EMG parameters from each window. (c) Removing outliers and applying smoothing to the EMG parameters. (d) Performing linear regression and comparing the initial and final data points of the EMG parameters. (e) Computing evaluation metrics. The same steps were applied to the ECG data, but evaluation metrics were computed only from heart rate.

In the present study, signal processing was performed using custom MATLAB R2023b (The MathWorks, Natick, MA, USA) routines (Figure 2). The EMG signals were band-pass filtered from 10 to 450 Hz using a fourth-order zero-lag Butterworth filter and notch filtered at 50 Hz, while the raw ECG signal was filtered using a second-order zero-lag Butterworth filter ranging from 0.5 to 40 Hz and similarly notch filtered at 50 Hz. The dataset included periods of rest (i.e., no muscle activation) and task-related activity (i.e., muscle activation). The activation phase was defined as the time between the initial shoulder lift (i.e., task start), followed by 6 min of sustained activity, and the lowering of the shoulder (i.e., task end). For each participant and condition (NO EXO, EXO L, EXO M, EHO H), the activation phases for all muscles are identified using the right AD muscle as reference due to the movement symmetry between left and right arms. Pre-processed signals were analyzed according to the following steps (Figure 2a).

First, the activation phase in both EMG and ECG signals was divided into 5-s epochs (Bosch et al., 2009). Within each 5-s epoch, a 4-s sliding window (80% overlap) was used. For each EMG epoch, a Hanning window was applied to reduce spectral leakage before calculating the power spectrum using fast Fourier transform, which was then converted to a single-sided power spectrum to compute the median frequency (MDF) (Lotz et al., 2009) over a sliding window. For the ECG signal, the R peaks of each QRS complex were detected within each epoch. The R-R interval, which measures the time distance between two consecutive R waves in adjacent QRS complexes, was then determined (Halomoan et al., 2023). The ECG-based parameters, namely, HR and HRV metrics – root mean square of successive differences (RMSSD) and ratio of low-frequency power to high-frequency power (LF/HF) – were calculated using the same sliding windows as done for the MDF. To extract LF/HF ratio, the R-R data were first interpolated and evenly sampled at 4 Hz. Welch’s power spectral density was then applied to determine the LF/HF ratio. From the power spectral density (PSD) estimates, the powers for the defined frequency bands LF (0.04–0.15 Hz) and HF (0.15–0.4 Hz) were computed (Vaishali et al., 2020).

To remove outliers from both the EMG and ECG parameters, the Hampel identifier filter was applied (Bhowmik et al., 2017): for each outlier, the filter computes the median of a window composed of the outlier sample and 50 surrounding samples, 25 per side. The resulting estimates were then smoothed using a moving average filter with a window size of 50 samples (20 Hz), providing a smoother representation of the EMG and ECG signal parameter behaviors during task execution.

Once MDF and HR profiles were available, we computed the (i) the *relative change* ( 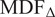 and 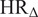 ) along the task execution. This was calculated by first computing the median values of the MDF and HR over the first and last 25 s of the task. The relative change was then computed as the percentage difference between the median values at the end ( 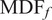 ) and the start ( 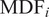 ) of the task using the initial median value ( 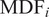 ) as the reference, and (ii) the slope and intercept of a linear regressor are computed by fitting a straight line to the set of data points using the method of least squares regression as shown in Figure 2b (Yoon and Shin, 2024).

Negative 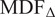 values indicate increased muscle fatigue, whereas positive 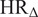 values indicate increasing cardiac effort over the course of the trial. The slope of the regression line described the rate of change over time in both MDF and HR signals. A negative slope in the MDF profile 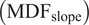 is an indicator of muscle fatigue (Yoon and Shin, 2024), whereas a positive slope of the HR signal ( 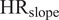 ) indicates increasing physical demand. Conversely, a negative 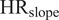 indicates a person is recovering from previous exertion. The intercept was a measure of the estimated initial value for both MDF ( 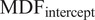 ) and HR ( 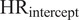 ) signals at the beginning of each trial representing baseline values for MDF and HR signals (Sood et al., 2007; Lamers et al., 2020).

The extracted MDF and HR metrics for each subject, condition (NO EXO, EXO L, EXO M, EXO H), and muscle (AD and PD) were aggregated across all participants as median and interquartile range.

Finally, HRV metrics, including RMSSD and LF/HF ratio, were computed from the ECG data. RMSSD primarily reflects parasympathetic activity (Shaffer and Ginsberg, 2017): lower RMSSD suggests reduced vagal tone, indicating a shift toward sympathetic dominance and potential cardiovascular fatigue. The LF/HF ratio is often used to assess the balance between sympathetic and parasympathetic modulation (Vaishali et al., 2020): higher LF/HF ratio suggests increased sympathetic dominance, which is commonly linked to greater physiological strain and fatigue.

### 2.4. Statistical analysis

Statistical analysis was performed with custom MATLAB R2023b routines to evaluate the effect of different assistance levels on the computed metrics. For each condition, the normality of the computed metrics was assessed using the Shapiro-Wilk test. Since relative change, slope, and intercept for both MDF and HR were not normally distributed, non-parametric one-way repeated-measures ANOVAs (Friedman test) were applied to examine differences. The test was conducted separately for each muscle, each side, and for each parameter computed from MDF and HR signals. When applicable, post-hoc paired comparisons were assessed using the Wilcoxon signed-rank test. All statistical analyses were conducted using a significance level *α* = 0.05.

## 3. Results

This section presents the results examining how different levels of anti-gravitational support provided by the exoskeleton influenced peripheral fatigue in the shoulder muscles and central cardiovascular fatigue.

### 3.1. Muscle fatigue indicators

**Figure 3.**
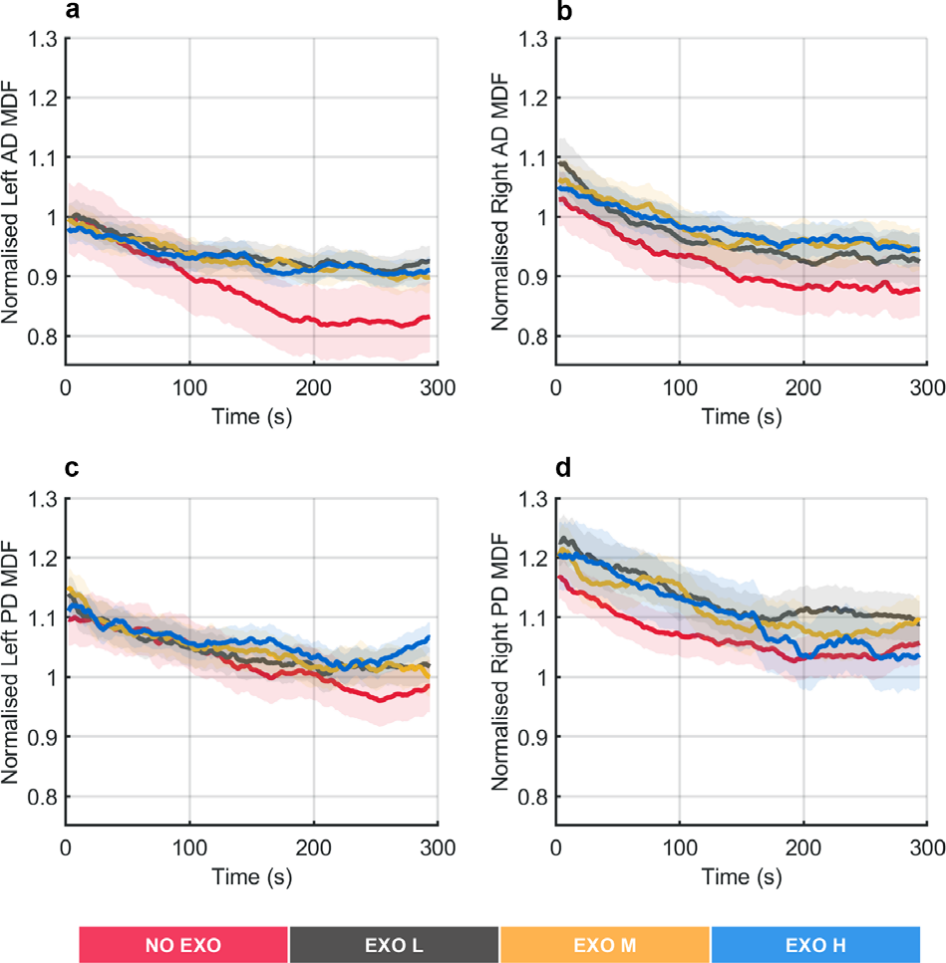
Normalized median frequency (MDF) profiles averaged across all participants during the overhead screwing experiment, shown for the left and right anterior deltoid (AD) and the left and right posterior deltoid (PD) muscles. Shaded area represents standard deviation, while the lines indicate the moving average applied to the MDF values.

Figure 3 shows the MDF profiles normalized using MDF computed from MVC values, averaged across all participants, for left and right shoulder muscles during the execution of the overhead screwing task.

A substantial decrease in MDF over time was observed for the AD and PD muscles in the NO EXO condition. In contrast, when anti-gravitational support was provided by the exoskeleton, the decrease over time in MDF was smaller.

**Figure 4.**
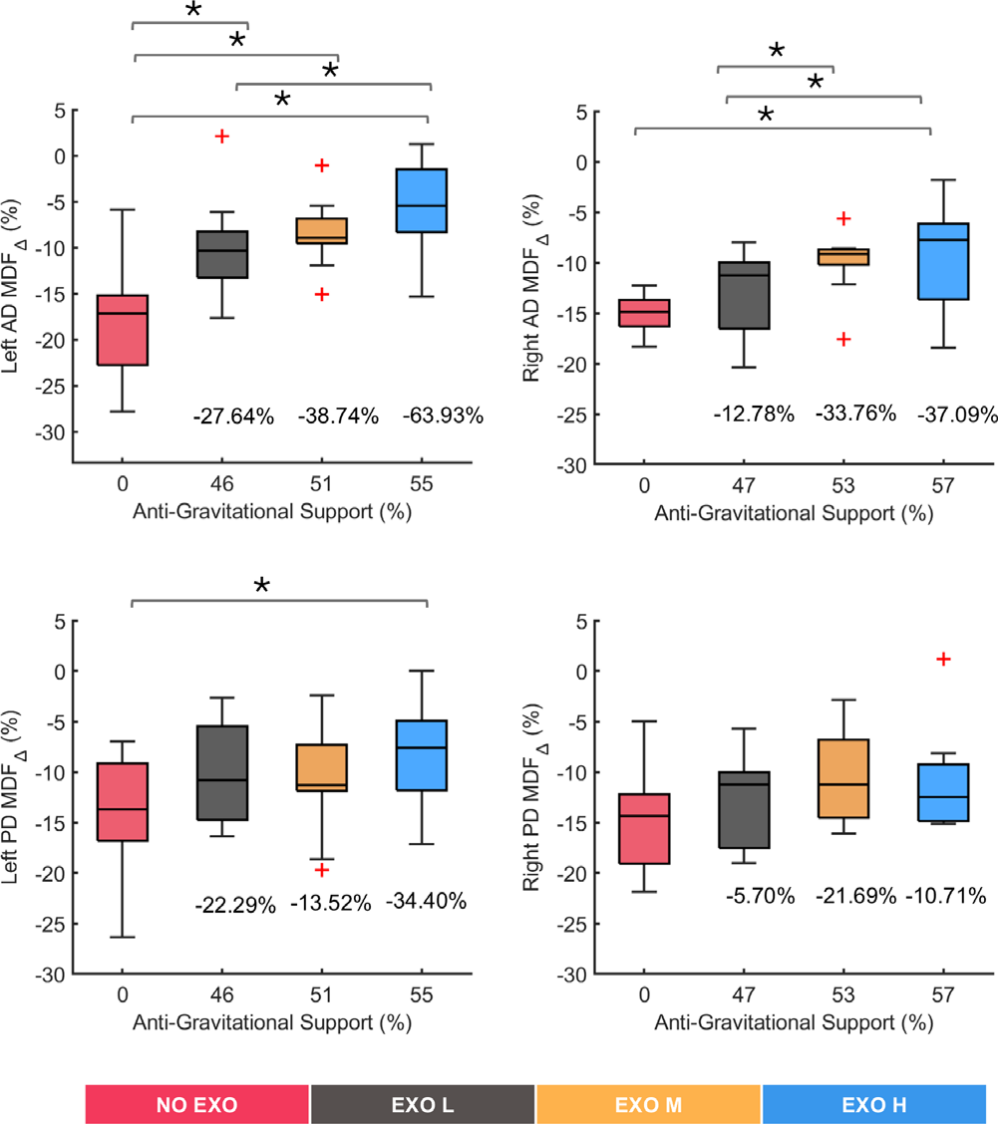
Relative change in median frequency (MDF). Results are shown for left and right anterior deltoid (AD) and left and right posterior deltoid (PD) muscles. Anti-gravitational support values provided by the exoskeleton are reported as median values across all participants. Plus marks represent the outlier in the data. Bars represent the change across conditions, and asterisks mark statistically significant differences between conditions. Percentage differences are reported compared to the no exoskeleton (NO EXO) condition.

Figure 4 shows the 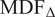 values for all muscles in all tested conditions. Significant differences were found in the left AD muscle (*χ^2^* (3) = 16.68, *p* = 0.0008) and right AD muscle (*χ^2^* (3) = 12.96, *p* = 0.0047), showing that both AD muscle’s fatigue was affected using the exoskeleton. Post hoc pairwise comparison between conditions showed significant differences between the NO EXO condition and all EXO conditions, up to around 64% in the EXO H condition (*p* = 0.0020) for the left-side muscles; conversely, for the right side, significant difference compared to the NO EXO was found only in the EXO H condition of around 37% (*p* = 0.0098). Significant differences were also found between EXO L and EXO H conditions in both left (*p* = 0.0273) and right (*p* = 0.0371) AD muscles.

Significant differences were found in the left PD muscle (*χ^2^* (3) = 9, *p* = 0.0293), whereas no significant differences were observed in the right PD muscle (*χ^2^* (3) = 3.24, *p* = 0.3561), showing small effects of the exoskeleton on the PD muscles. Post hoc pairwise differences in left PD were smaller compared to the AD muscle, across conditions, with only the EXO H condition showing significant variation with respect to the NO EXO (*p* = 0.0273).

Overall, the above results suggested that wearing the exoskeleton significantly influenced muscle fatigue in the AD muscles, particularly at higher support levels, while its effect on PD muscles was not pronounced, with the right PD muscle showing no significant change.

**Figure 5.**
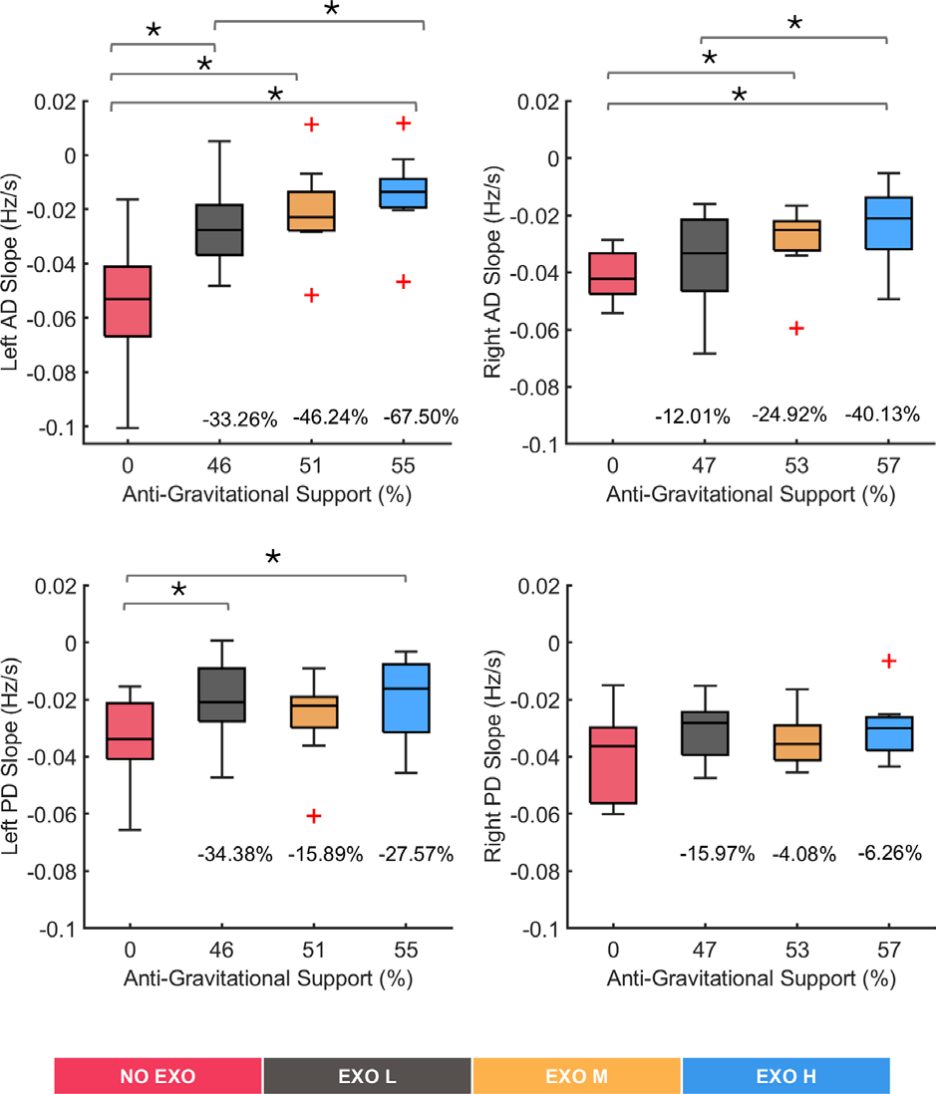
Median frequency slope values for all tested conditions (NO EXO, EXO L, EXO M, EXO H) for left and right anterior deltoid (AD) and left and right posterior deltoid (PD) muscles. Anti-gravitational support values provided by the exoskeleton are reported as median values across all participants. Bars represent the change across conditions, and asterisks mark statistically significant differences between conditions. Percentage differences are reported compared to the no-exoskeleton condition.

Figure 5 shows the 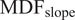 across different experimental conditions. The linear regression analysis showed a decreasing trend in MDF values across all conditions in each muscle, with R-squared (R^2^) values ranging between 0.9 and 0.4. The Friedman test revealed significant differences in muscle activity with exoskeleton support. Specifically, left AD muscle (*χ* ^2^ (3) = 12.48, *p* = 0.0059), right AD muscle (*χ* ^2^ (3) = 8.28, *p* = 0.0406), and left PD muscle (*χ* ^2^ (3) = 7.56, *p* = 0.056) showed significant changes, indicating that exoskeleton support influenced these muscles. In contrast, the right PD muscle showed no significant changes (*χ* ^2^ (3) = 2.64, *p* = 0.4505), suggesting no effects of the exoskeleton on this muscle. The differences between NO EXO and EXO H were up to 67% (*p* = 0.0039) and 40% (*p* = 0.0098) for left and right AD muscles, respectively, and 27% (*p* = 0.0488) for left PD muscles.

**Figure 6.**
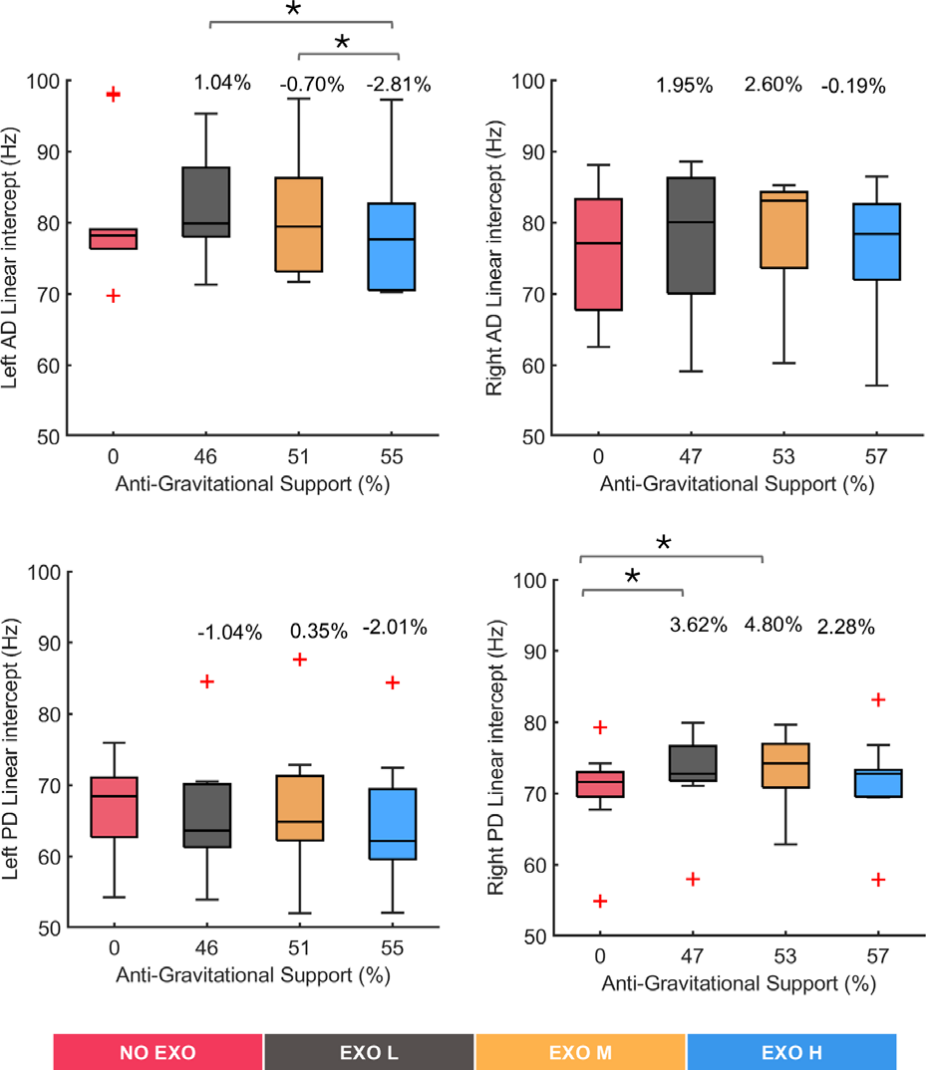
Median frequency intercept values for all tested conditions (NO EXO, EXO L, EXO M, EXO H) for left and right anterior deltoid (AD) and left and right posterior deltoid (PD) muscles. Anti-gravitational support values provided by the exoskeleton are reported as median values across all participants. Bars represent the change across conditions, and asterisks mark statistically significant differences between conditions. Percentage differences are reported compared to the NO EXO condition.

Figure 6 shows 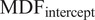 values reflecting MDF at the start of each task execution across the four tested conditions. Significant differences were observed in the left AD (*χ^2^* (3) = 9.48, *p* = 0.024) and the right PD (*χ^2^* (3) = 9.72, *p* = 0.0211), whereas no significant changes were found in right AD (*χ^2^* (3) = 4.08, *p* = 0.253) and left PD (*χ^2^* (3) = 5.64, *p* = 0.135). Post hoc pairwise comparison between conditions showed significant differences in the left AD muscle between the EXO L and EXO H conditions (*p* = 0.0137) and between EXO M and EXO H (*p* = 0.0020). In the right PD muscle, significant differences were identified between the NO EXO and EXO L conditions (*p* = 0.0039), with a difference of approximately 3.62%, and between NO EXO and EXO M (*p* = 0.0371), with a difference of around 4.80%. However, such differences were less than 5%, indicating that, on average, all conditions started from comparable MDF values.

### 3.2. Cardiovascular fatigue indicators

**Figure 7.**
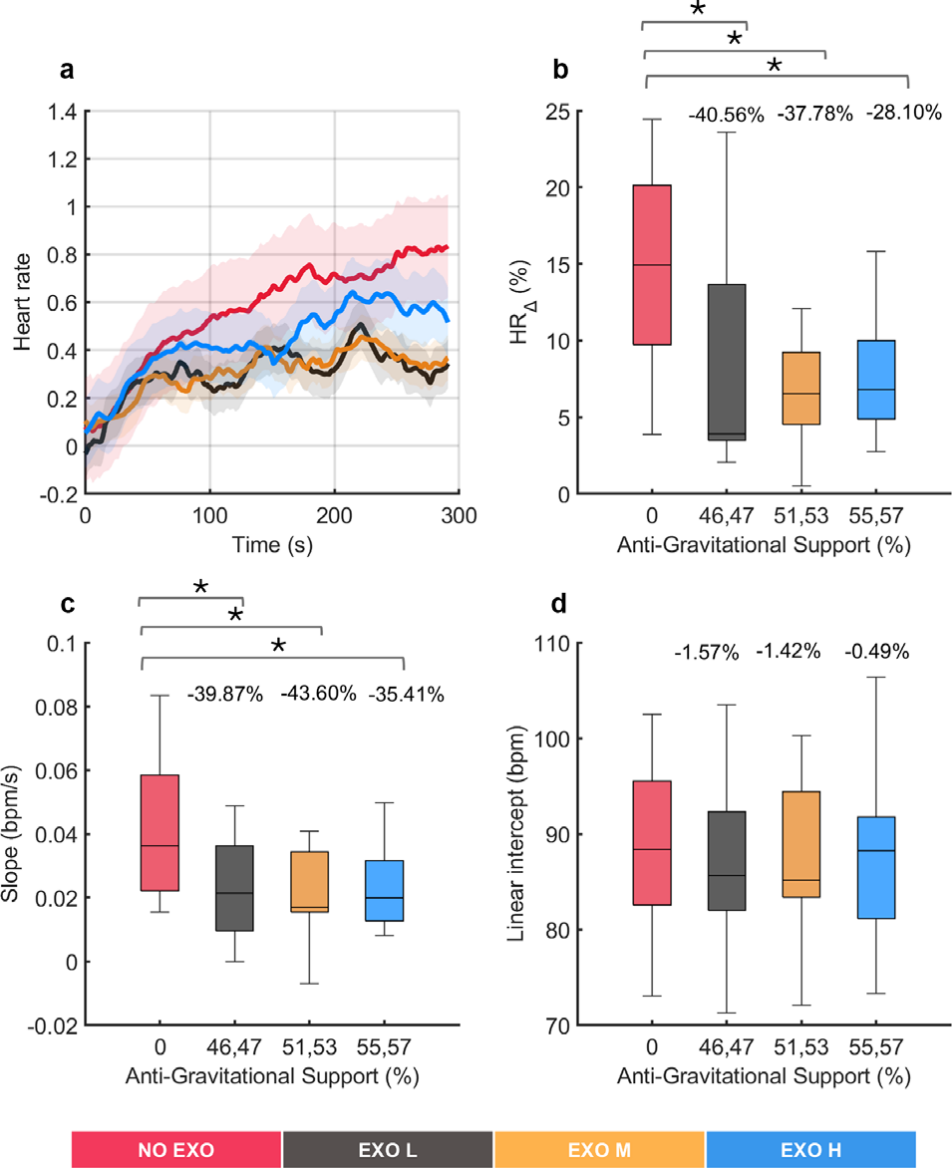
Analysis of cardiovascular fatigue under no exoskeleton (NO EXO) and exoskeleton conditions (EXO L, EXO M, and EXO H). (a) Normalized heart rate (HR) profiles (as %) averaged across all participants during the overhead screwing experiment for all tested conditions. Shaded area represents the standard deviation, while the lines indicate the moving average applied to the HR data. Evaluation metrics of HR under varying levels of anti-gravitational support are shown aggregated across all subjects: (b) relative change in HR, (c) slope of the linear fit on HR, and (d) intercept of the linear fit on HR. Anti-gravitational support values provided by the exoskeleton are reported as median values (both sides). Bars represent the change across conditions and asterisks mark statistically significant differences between conditions. Percentage differences are reported compared to the NO EXO condition.

ECG parameters for all conditions were normalized using baseline ECG data from the NO EXO condition and averaged across all participants over time. The results of HR for all tested conditions are presented in Figure 7(a) while aggregated data from of all participants are shown in Figure 7(b–d).

Throughout the task, an increasing trend was observed in HR values, with R^2^ values ranging from 0.5 to 0.7 across all conditions in a regression analysis. Significant differences were observed in 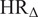 (*χ^2^* (3) = 8.04, *p* = 0.0452) and in 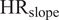 (*χ^2^* (3) = 10.32, *p* = 0.016), showing that HR activity was affected using the exoskeleton. Post hoc pairwise comparison between conditions revealed, in both metrics ( 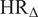 and 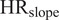 ) significant differences were found between all EXO conditions and the NO EXO condition, whereas no significant differences were found among the EXO conditions. As regards to the 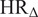 , significant differences were found compared to NO EXO and all EXO conditions, up to around 40% (*p* < 0.0273), and for 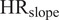 , significant differences between NO EXO condition and all EXO conditions were observed up to around 43% (*p* < 0.0098). This indicated that wearing an exoskeleton affected 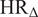 and 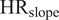 , compared to not wearing one, but all EXO conditions had a similar impact. Conversely, the 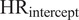 showed no significant differences (*χ^2^* (3) = 2.04, *p* = 0.564), indicating that, on average, all conditions started from comparable HR values.

**Figure 8.**
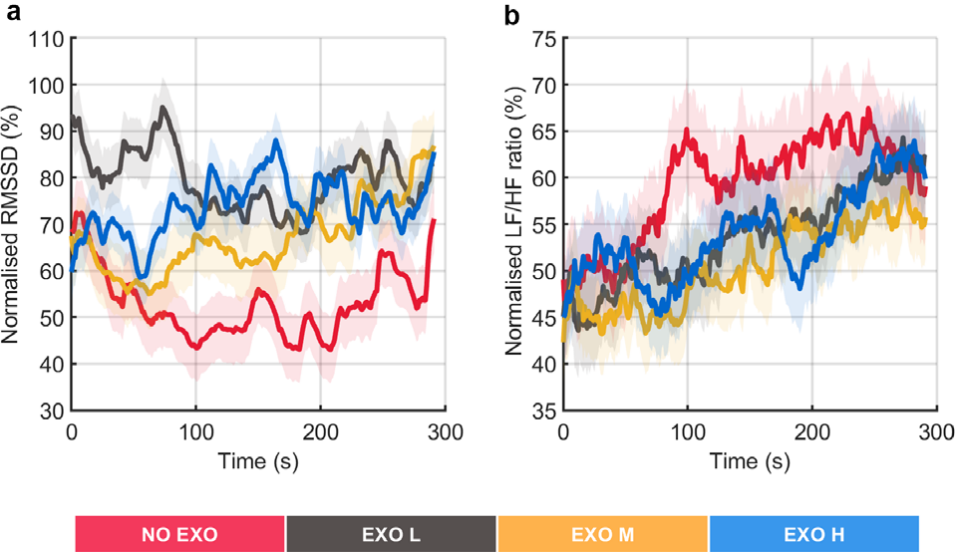
Representation of heart rate variability. The figure shows normalized root mean square of successive differences (RMSSD) and low-frequency to high-frequency power ratio (LF/HF) profiles (as %) averaged across all participants (a–b). The shaded area represents the standard deviation, while the lines indicate the moving average applied to the data.

Figure 8 presents HRV metrics, including RMSSD and LF/HF ratio, for exoskeleton-assisted and no exoskeleton conditions during overhead screwing task. Figure 8a does not show a visible trend along the time in the RMSSD metric regardless of the assisted conditions, suggesting higher parasympathetic dominance, which indicates relaxation and recovery. In contrast, Figure 8b shows LF/HF ratio, which increases over time in all conditions, indicating a shift toward sympathetic dominance, suggesting a state of stress or fatigue.

## 4. Discussion

The findings of this study revealed that through exoskeleton use the participants significantly reduced muscle fatigue indicators compared to the condition in which the exoskeleton was not worn. This result was particularly relevant for the AD muscles, which are the main muscles involved in keeping the arms raised overhead to perform the task. Notably, changes in the peripheral muscle fatigue differed significantly between the NO EXO condition, which showed a marked decrease in 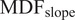 and 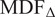 over time, and the conditions involving exoskeletal assistance (EXO L, EXO M, EXO H). However, the variations between different assistance levels, corresponding to varying amounts of anti-gravitational support, were less clear. This could be explained by individual differences in body size, shape, and proportions, which could lead to varying experiences of support effectiveness, making the results less consistent in MDF, HR, and HRV. In passive exoskeletons, the device typically supports only a fraction of the torque generated by gravity at the shoulder joint. As a result, individuals with larger anthropometry require a higher level of assistance to receive the same degree of anti-gravitational support as those with smaller anthropometry. Nevertheless, it can be observed that flexor muscles, in prolonged static exertion performed overhead, can benefit more from higher levels of anti-gravitational support in terms of reducing muscle fatigue. This result aligns with previous evidence obtained from EMG amplitude analysis, where EMG activation was found to achieve greater reduction when the higher assistance was provided (Grazi et al., 2020). Moreover, the results of this study highlighted a less clear effect on PD muscles, whose fatigue indicators seemed to be weakly affected by the amount of anti-gravitational support. This different behavior could be explained by the fact that anterior deltoids were primarily involved in keeping the arms raised in the overhead position, whereas posterior deltoids were involved in stabilizing the glenohumeral joint (Halder et al., 2000), thus potentially leading to a different influence on muscle fatigue. Another explanation may possibly be that the participants were right-handed, and they likely used their dominant arm more efficiently for the overhead screwing task. This efficiency may have reduced the need for stabilization from the PD muscle. In contrast, the non-dominant arm, being less skilled, may have relied more on the PD muscle for stability, leading to greater fatigue. Furthermore, the exoskeleton’s assistance in reducing agonist muscle activity (AD) may have lessened the demand on the PD muscle. Its role in stabilizing the shoulder during sustained, intense effort was likely compensated by other muscle groups. Heart rate metrics ( 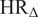 and 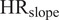 ) and HRV metrics (RMSSD, LF/HF ratio) were used in this study to investigate the effect of exoskeleton on overall cardiovascular fatigue. Our analysis showed that using the exoskeleton reduced cardiovascular load compared to the NO EXO condition, as demonstrated by the substantial lower values of 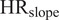 and 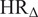 along with changes in HRV metrics. Participants experienced higher stress without exoskeleton support, as indicated by lower HRV, suggesting greater physiological strain in the absence of assistance. Nevertheless, as in the case of muscle fatigue, the amount of assistance was uncorrelated with the estimated cardiovascular fatigue. Similar to Maurice et al. (2020) and Schmalz et al. (2019), who observed decreased up to 19 and 6% of the cardiovascular load when exoskeletons were used, this study confirms the cardiovascular benefits of exoskeletons but adds that variations in support levels did not substantially affect HR metrics. This could be attributed to the small sample size. Moreover, as previously discussed, from a methodological perspective, the three assistance levels corresponded to different percentages of anti-gravitational support for individual participants, which may have influenced the variability of HR data across conditions and, consequently, could explain the observed result.

Notably, the MDF, HR, and HRV exhibited similar trends in indicating physical fatigue as the level of assistance changed. However, this effect might be due to the relatively narrow range of peak torque across the different assistive conditions. Specifically, the peak torque ranged from approximately 4.5–6 N.m, corresponding to about 46–57% of anti-gravitational support at the shoulder joint. This limited range of support may have resulted in similar reductions in physical fatigue. Furthermore, these results are specific to the experimental task and protocol, the sample population, and the exoskeleton used in this study. Therefore, altering any of these factors could yield different outcomes, such as increasing the duration of each physical exertion or using an exoskeleton capable of spanning a wider range of assistive supports.

While the analysis of time-variant parameters has shown the potential effectiveness of the exoskeleton in reducing physical fatigue, the results related to the analysis of intercept values ( 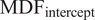 and 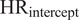 ) suggest that none of the experimental conditions were influenced by bias from prior fatigue. This indicates that participants began each trial in a rested state, both in terms of muscular and cardiovascular viewpoint. This behavior was ensured by allowing the participants to have adequate time to rest to avoid fatigue accumulation that could have been a source of unwanted variability in the data, potentially preventing their correct interpretation.

The findings of this study align with and build upon existing research on the effectiveness of shoulder OEs in reducing physical strain during overhead tasks. Prior studies have primarily focused on muscle activation in lab-based industry-inspired static/quasi-static (Huysamen et al., 2018; Maurice et al., 2020; Pacifico et al., 2020, 2022; Schmalz et al., 2019; Spada et al., 2017; Van Engelhoven et al., 2019) and repetitive/dynamic tasks. However, the exploration of muscular and cardiovascular fatigue remains relatively underexplored. While previous research widely demonstrated the advantages of using OEs when the objective is achieving the reduction of muscle activation, only a few studies adequately investigated how peripheral fatigue as well as global cardiovascular fatigue are affected using shoulder exoskeletons. De Bock and colleagues reported a 41% reduction in peripheral muscle fatigue with exoskeleton assistance showing that it can improve muscle endurance during prolonged tasks (De Bock et al., 2022); other studies observed how heart rate reduced with exoskeleton use (Maurice et al., 2020; Schmalz et al., 2019). Nevertheless, the evaluations performed in these studies did not consider how exoskeleton use specifically impacts fatigue progression over prolonged time use. Although such evaluation has not been performed for upper-limb OEs, a similar analysis has been conducted for back-support exoskeletons (Lotz et al., 2009; Lamers et al., 2020), where peripheral muscle fatigue indicators were assessed over the entire duration of physical exertion. However, this assessment was done only to compare fatigue when wearing/not wearing exoskeletons. With our study, we aimed at filling this gap found in previous research by evaluating how different assistance levels influenced physical fatigue. However, this research was conducted under controlled experimental conditions using quasi static, well-defined movements to evaluate the effects of the OE on physical fatigue. This approach minimized confounding factors from variable gestures, ensuring the rigor required for biomechanical studies. In this context, we believe our findings contribute to a better understanding of exoskeleton effectiveness. By monitoring fatigue parameters (HR and EMG activity), workers’ exposure to the physical demands can be minimized, reducing the risk of musculoskeletal injuries and enhancing overall safety (Dias et al., 2023). Indeed, a study by (Oglesby et al., 1989) described that a tense muscle can hold 15% of its maximum resistance indefinitely, 50% for 1 min, but maximum force for only 6–7 s. This demonstrates how prolonged force exertion can cause injury, particularly when workers exceed their physical limits.

Although the results of this study are valuable for assessing the impact of OEs, there are some limitations. First, the study was conducted on a small sample of healthy young individuals, which does not reflect the actual working population and could limit the statistical power of the findings. The small sample size may have also restricted the ability to detect subtle differences between assistive conditions. Additionally, in the study only two shoulder muscles were considered. However, this analysis could be extended also to other muscles for a more comprehensive analysis while also considering variations in participants’ anthropometry, which may influence the effectiveness of exoskeleton support.

Future work shall address the limitations to enhance the generalizability of the present results to more realistic conditions, namely, with a large population of actual workers in real-work conditions. Additionally, future studies should consider conducting a priori power analysis to determine the optimal sample size based on the desired power of the study. Furthermore, implementing real-time monitoring strategies and integrating technological tools is essential for helping workers manage varying levels of fatigue during tasks, while several methods have been proposed to allow the real-time monitoring of physical fatigue at work, such as the use of smart wearable devices (Moshawrab et al., 2022). To the best of the authors’ knowledge, none of them can be concurrently used to provide a physical intervention to reduce the levels of physical fatigue experienced by workers. OEs have the potential to be used as tools that workers can wear to cope with increasing physical demand due to heavy or prolonged work tasks (De Looze et al., 2016). Moreover, when dealing with active or semi-active exoskeletons, we can consider integrating these devices with external sensing apparatuses designed to measure physical fatigue in real time. A further research step could be the development of adaptive algorithms that can adjust the level of assistance provided by the exoskeleton according to the worker’s physical fatigue in real time: this could potentially allow to study how fatigue can be modulated through adaptive exoskeleton support.

## 5. Conclusion

This study contributed to understanding how different levels of semi-active exoskeleton support influence peripheral muscle and global cardiovascular fatigue. Our findings underscore the potential of OEs as ergonomic tools that promote physical well-being, reduce fatigue, and support sustainable work practices in overhead occupational settings.

## Data Availability

Data can be made available to interested researchers upon reasonable request by email to the corresponding author.

## References

[r1] Antwi-Afari MF, Li H, Edwards DJ, Pärn EA, Seo J and Wong AYL (2017) Biomechanical analysis of risk factors for work-related musculoskeletal disorders during repetitive lifting task in construction workers. Automation in Construction 83, 41–47. 10.1016/j.autcon.2017.07.007.

[r2] Bangaru SS, Wang C and Aghazadeh F (2022) Automated and continuous fatigue monitoring in construction workers using forearm EMG and IMU wearable sensors and recurrent neural network. Sensors 22(24), 9729. 10.3390/s22249729. 36560096 PMC9786306

[r3] Bevan S (2015) Economic impact of musculoskeletal disorders (MSDs) on work in Europe. Best Practice & Research Clinical Rheumatology 29(3), 356–373. 10.1016/j.berh.2015.08.002. 26612235

[r4] Bhowmik S, Jelfs B, Arjunan SP and Kumar DK (2017) Outlier removal in facial surface electromyography through Hampel filtering technique. 2017 IEEE Life Sciences Conference (LSC), 258–261. IEEE. 10.1109/LSC.2017.8268192.

[r5] Bosch T, De Looze MP, Kingma I, Visser B and Van Dieën JH (2009) Electromyographical manifestations of muscle fatigue during different levels of simulated light manual assembly work. Journal of Electromyography and Kinesiology 19(4), e246–e256. 10.1016/j.jelekin.2008.04.014. 18586520

[r6] Chen Y, Liu M, Zhou J, Bao D, Li B and Zhou J (2023) Acute effects of fatigue on cardiac autonomic nervous activity. Journal of Sports Science and Medicine, 22(4), 806–815. 10.52082/jssm.2023.806. 38045744 PMC10690502

[r7] Cifrek M, Medved V, Tonković S and Ostojić S (2009) Surface EMG based muscle fatigue evaluation in biomechanics. Clinical Biomechanics 24(4), 327–340. 10.1016/j.clinbiomech.2009.01.010. 19285766

[r8] Cifrek M, Tonković S and Medved V (2000) Measurement and analysis of surface myoelectric signals during fatigued cyclic dynamic contractions. Measurement 27(2), 85–92. 10.1016/S0263-2241(99)00059-7.

[r9] Crea S, Beckerle P, De Looze M, De Pauw K, Grazi L, Kermavnar T, Masood J, O’Sullivan LW, Pacifico I, Rodriguez-Guerrero C, Vitiello N, Ristić-Durrant D and Veneman J (2021) Occupational exoskeletons: A roadmap toward large-scale adoption. Methodology and challenges of bringing exoskeletons to workplaces. Wearable Technologies 2, e11. 10.1017/wtc.2021.11. 38486625 PMC10936259

[r10] De Bock S, Rossini M, Lefeber D, Rodriguez-Guerrero C, Geeroms J, Meeusen R and De Pauw K (2022) An occupational shoulder exoskeleton reduces muscle activity and fatigue during overhead work. IEEE Transactions on Biomedical Engineering 69(10), 3008–3020. 10.1109/TBME.2022.3159094. 35290183

[r11] De Kok J, Vroonhof P, Snijders J, Roullis G, Clarke M, Peereboom K and van Dorst P (2019) Work–related musculoskeletal disorders – Prevalence, costs and demographics in the EU. Luxembourg: Publications Office of the European Union https://data.europa.eu/doi/10.2802/66947.

[r12] De Looze MP, Bosch T, Krause F, Stadler KS and O’Sullivan LW (2016) Exoskeletons for industrial application and their potential effects on physical work load. Ergonomics 59(5), 671–681. 10.1080/00140139.2015.1081988. 26444053

[r13] De Luca CJ (1984) Myoelectrical manifestations of localized muscular fatigue in humans. Critical Reviews in Biomedical Engineering 11(4), 251–279. 6391814

[r14] Dias M, Silva L, Folgado D, Nunes ML, Cepeda C, Cheetham M and Gamboa H (2023) Cardiovascular load assessment in the workplace: A systematic review. International Journal of Industrial Ergonomics 96, 103476. 10.1016/j.ergon.2023.103476.

[r15] Dickerson CR, Brookham RL and Chopp JN (2011) The working shoulder: Assessing demands, identifying risks, and promoting healthy occupational performance. Physical Therapy Reviews 16(5), 310–320. 10.1179/1743288X11Y.0000000032.

[r16] Dideriksen JL, Farina D and Enoka RM (2010) Influence of fatigue on the simulated relation between the amplitude of the surface electromyogram and muscle force. Philosophical Transactions of the Royal Society A: Mathematical, Physical and Engineering Sciences 368(1920), 2765–2781. 10.1098/rsta.2010.0094. 20439272

[r17] Feng W, Zeng K, Zeng X, Chen J, Peng H, Hu B and Liu G (2023) Predicting physical fatigue in athletes in rope skipping training using ECG signals. Biomedical Signal Processing and Control 83, 104663. 10.1016/j.bspc.2023.104663.

[r18] Fisher JP and Secher NH (2019) Regulation of heart rate and blood pressure during exercise in humans. In Muscle and Exercise Physiology. Elsevier, Amsterdam, Netherlands. pp. 541–560. 10.1016/B978-0-12-814593-7.00024-4

[r19] Gandevia SC (2001) Spinal and supraspinal factors in human muscle fatigue. Physiological Reviews 81(4), 1725–1789. 10.1152/physrev.2001.81.4.1725. 11581501

[r20] Gatti UC, Migliaccio GC, Bogus SM and Schneider S (2014) An exploratory study of the relationship between construction workforce physical strain and task level productivity. Construction Management and Economics 32(6), 548–564. 10.1080/01446193.2013.831463.

[r21] Gawron VJ, French J and Funke D (2001) An overview of fatigue. In Hancock, P.A. and Desmond, P.A., Stress, Workload, and Fatigue, Lawrence Erlbaum Associates Publishers pp. 581–595.

[r22] Grazi L, Trigili E, Fiore M, Giovacchini F, Sabatini AM, Vitiello N and Crea S (2024) Passive shoulder occupational exoskeleton reduces shoulder muscle coactivation in repetitive arm movements. Scientific Reports 14(1), 27843. 10.1038/s41598-024-78090-2. 39537722 PMC11561117

[r23] Grazi L, Trigili E, Proface G, Giovacchini F, Crea S and Vitiello N (2020) Design and experimental evaluation of a semi-passive upper-limb exoskeleton for workers with motorized tuning of assistance. IEEE Transactions on Neural Systems and Rehabilitation Engineering 28(10), 2276–2285. 10.1109/TNSRE.2020.3014408. 32755865

[r24] Halder AM, Itoi E and An K-N (2000) Anatomy and biomechanics of the shoulder. Orthopedic Clinics of North America 31(2), 159–176. 10.1016/S0030-5898(05)70138-3. 10736387

[r25] Halomoan J, Ramli K, Sudiana D, Gunawan TS and Salman M (2023) ECG-based driving fatigue detection using heart rate variability analysis with mutual information. Information 14(10), 539. 10.3390/info14100539.

[r26] Hermens HJ, Freriks B, Disselhorst-Klug C and Rau G (2000) Development of recommendations for SEMG sensors and sensor placement procedures. Journal of Electromyography and Kinesiology 10(5), 361–374. 10.1016/S1050-6411(00)00027-4. 11018445

[r27] Huysamen K, Bosch T, de Looze M, Stadler KS, Graf E and O’Sullivan LW (2018) Evaluation of a passive exoskeleton for static upper limb activities. Applied Ergonomics 70, 148–155. 10.1016/j.apergo.2018.02.009. 29866305

[r28] Lamers EP, Soltys JC, Scherpereel KL, Yang AJ and Zelik KE (2020) Low-profile elastic exosuit reduces back muscle fatigue. Scientific Reports 10(1), 15958. 10.1038/s41598-020-72531-4. 32994427 PMC7524767

[r29] Larsson B, Søgaard K and Rosendal L (2007) Work related neck–shoulder pain: A review on magnitude, risk factors, biochemical characteristics, clinical picture and preventive interventions. Best Practice & Research Clinical Rheumatology 21(3), 447–463. 10.1016/j.berh.2007.02.015. 17602993

[r30] Lotz CA, Agnew MJ, Godwin AA and Stevenson JM (2009) The effect of an on-body personal lift assist device (PLAD) on fatigue during a repetitive lifting task. Journal of Electromyography and Kinesiology 19(2), 331–340. 10.1016/j.jelekin.2007.08.006. 18055220

[r31] Maurice P, Camernik J, Gorjan D, Schirrmeister B, Bornmann J, Tagliapietra L, Latella C, Pucci D, Fritzsche L, Ivaldi S and Babic J (2020) Objective and subjective effects of a passive exoskeleton on overhead work. IEEE Transactions on Neural Systems and Rehabilitation Engineering 28(1), 152–164. 10.1109/TNSRE.2019.2945368. 31581086

[r32] Monica L, Sara Anastasi S and Francesco Draicchio F (2020) Occupational exoskeletons: Wearable robotic devices to prevent work-related musculoskeletal disorders in the workplace of the future. Luxembourg: European Agency for Safety and Health at Work. Available at: https://osha.europa.eu/sites/default/files/MSDs_Occupational_exoskeletons_wearable_devices.pdf

[r33] Moshawrab M, Adda M, Bouzouane A, Ibrahim H and Raad A (2022) Smart Wearables for the detection of occupational physical fatigue: A literature review. Sensors 22(19), 7472. 10.3390/s22197472. 36236570 PMC9573761

[r34] Moyon A, Poirson E and Petiot J-F (2018) Experimental study of the physical impact of a passive exoskeleton on manual sanding operations. Procedia CIRP 70, 284–289. 10.1016/j.procir.2018.04.028.

[r53] O’Neill, C. and Panuwatwanich, K. 2013. The impact of fatigue on labour productivity: Case study of dam construction project in Queensland. Proceedings of the Fourth International Conference on Engineering, Project and Production Management. Bangkok, Thailand: EPPM. Available at: https://www.researchgate.net/publication/263173988

[r35] Oglesby CH, Parker HW and Howell GA (1989) Productivity Improvement in Construction. New York: McGraw-Hill

[r36] Pacifico I, Parri A, Taglione S, Sabatini AM, Violante FS, Molteni F, Giovacchini F, Vitiello N and Crea S (2022) Exoskeletons for workers: A case series study in an enclosures production line. Applied Ergonomics 101, 103679. 10.1016/j.apergo.2022.103679. 35066399

[r37] Pacifico I, Scano A, Guanziroli E, Moise M, Morelli L, Chiavenna A, Romo D, Spada S, Colombina G, Molteni F, Giovacchini F, Vitiello N and Crea S (2020) An experimental evaluation of the proto-MATE: A novel ergonomic upper-limb exoskeleton to reduce workers’ physical strain. IEEE Robotics & Automation Magazine 27(1), 54–65. 10.1109/MRA.2019.2954105.

[r38] Parent-Thirion A, Vermeylen G, Wilkens M, Biletta I and Pot FD (2017) Towards the high road of workplace innovation in Europe? An illustration of the usefulness of the dataset of the European working conditions survey. In Oeij P, Rus D and Pot FD (eds), Workplace Innovation: Theory, Research and Practice. Springer International Publishing, Cham, Switzerland pp. 261–277. 10.1007/978-3-319-56333-6_16

[r39] Ramella G, Grazi L, Giovacchini F, Trigili E, Vitiello N and Crea S (2024) Evaluation of antigravitational support levels provided by a passive upper-limb occupational exoskeleton in repetitive arm movements. Applied Ergonomics 117, 104226. 10.1016/j.apergo.2024.104226. 38219374

[r40] Schmalz T, Schändlinger J, Schuler M, Bornmann J, Schirrmeister B, Kannenberg A and Ernst M (2019) Biomechanical and metabolic effectiveness of an industrial exoskeleton for overhead work. International Journal of Environmental Research and Public Health 16(23), 4792. 10.3390/ijerph16234792. 31795365 PMC6926884

[r41] Shaffer F and Ginsberg JP (2017) An overview of heart rate variability metrics and norms. Frontiers in Public Health 5, 258. 10.3389/fpubh.2017.00258. 29034226 PMC5624990

[r42] Sood D, Nussbaum MA and Hager K (2007) Fatigue during prolonged intermittent overhead work: Reliability of measures and effects of working height. Ergonomics 50(4), 497–513. 10.1080/00140130601133800. 17575711

[r43] Spada S, Ghibaudo L, Gilotta S, Gastaldi L and Cavatorta MP (2017) Investigation into the applicability of a passive upper-limb exoskeleton in automotive industry. Procedia Manufacturing 11, 1255–1262. 10.1016/j.promfg.2017.07.252.

[r44] Theurel J and Desbrosses K (2019) Occupational exoskeletons: Overview of their benefits and limitations in preventing work-related musculoskeletal disorders. IISE Transactions on Occupational Ergonomics and Human Factors 7(3–4), 264–280. 10.1080/24725838.2019.1638331.

[r45] Tran Y, Wijesuriya N, Tarvainen M, Karjalainen P and Craig A (2009) The relationship between spectral changes in heart rate variability and fatigue. Journal of Psychophysiology 23(3), 143–151. 10.1027/0269-8803.23.3.143.

[r46] Umer W, Li H, Yantao Y, Antwi-Afari MF, Anwer S and Luo X (2020) Physical exertion modeling for construction tasks using combined cardiorespiratory and thermoregulatory measures. Automation in Construction 112, 103079. 10.1016/j.autcon.2020.103079.

[r47] Vaishali B, Amalan S, Preejith SP, Joseph J and Sivaprakasam M (2020) HRV based stress assessment of individuals in a work environment. 2020 IEEE International Symposium on Medical Measurements and Applications (MeMeA), 1–6. 10.1109/MeMeA49120.2020.9137299.

[r48] Van Der Have A, Rossini M, Rodriguez-Guerrero C, Van Rossom S and Jonkers I (2022) The Exo4Work shoulder exoskeleton effectively reduces muscle and joint loading during simulated occupational tasks above shoulder height. Applied Ergonomics 103, 103800. 10.1016/j.apergo.2022.103800. 35598416

[r49] Van Engelhoven L, Poon N, Kazerooni H, Rempel D, Barr A and Harris-Adamson C (2019) Experimental evaluation of a shoulder-support exoskeleton for overhead work: Influences of peak torque amplitude, task, and tool mass. IISE Transactions on Occupational Ergonomics and Human Factors 7(3–4), 250–263. 10.1080/24725838.2019.1637799.

[r50] Vasquez-Bonilla AA, Yáñez-Sepúlveda R, Tuesta M, Martin EB-S, Monsalves-Álvarez M, Olivares-Arancibia J, Duclos-Bastías D, Recabarren-Dueñas C and Alacid F (2024) Acute fatigue impairs heart rate variability and resting muscle oxygen consumption kinetics. Applied Sciences 14(20), 9166. 10.3390/app14209166.

[r51] Wan J, Qin Z, Wang P, Sun Y and Liu X (2017) Muscle fatigue: General understanding and treatment. Experimental & Molecular Medicine 49(10), e384–e384. 10.1038/emm.2017.194. 28983090 PMC5668469

[r52] Yoon W and Shin G (2024) Muscle fatigue tracking during dynamic elbow flexion-extension movements with a varying hand load. Applied Ergonomics 116, 104217. 10.1016/j.apergo.2023.104217. 38160628

